# Diffuse Hair Loss Induced by Sertraline Use

**DOI:** 10.1155/2015/703453

**Published:** 2015-09-17

**Authors:** Yüksel Kıvrak, İbrahim Yağcı, Mehmet Fatih Üstündağ, Halil Özcan

**Affiliations:** ^1^Department of Psychiatry, Kafkas University Medical Faculty, Kars, Turkey; ^2^Department of Psychiatry, Atatürk University Medical Faculty, Erzurum, Turkey

## Abstract

Hair loss is a rare side effect of psychotropic drugs. The most related drug class with this side effect is the mood stabilizers. Studies reporting the sertraline-induced alopecia are limited in number. Sertraline is a potent antidepressant which inhibits the serotonin reuptake from the presynaptic terminals selectively. The reason for hair loss could not be elucidated completely. Psychotropic drugs are usually considered to lead to hair loss through influencing the telogen phase of hair follicle. This paper reports a 21-year-old male with diffuse hair loss induced by sertraline use and improved by quitting the drug. To the best of our knowledge, there are no other case reports on sertraline-induced alopecia within 2 weeks.

## 1. Introduction

Hair loss is among the side effects of psychotropic drugs [1]. The most related drug class with this side effect is the mood stabilizers [2]. Selective serotonin reuptake inhibitors (SSRIs) are commonly prescribed antidepressants for the treatments of major depressive disorder, obsessive compulsive disorder, panic disorder, generalized anxiety disorder, posttraumatic stress disorder, and many other psychiatric disorders [3]. There are a limited number of studies in the literature regarding the SSRI-induced alopecia.

Drug-induced hair loss generally diffuses with no scars and improves after quitting the drug. When drug-induced hair loss is suspected in a patient, other reasons for hair loss including hyperthyroidism, hypothyroidism, trichotillomania, hormonal disorders resulting from the hypothalamic-pituitary-gonadal axis disorders as well as iron, copper, and zinc deficiency, menopause, oral contraceptives, and use of other drugs (anticoagulants, anticonvulsants, antihypertensive agents, nonsteroidal anti-inflammatory drugs, and antiulcer drugs) should be considered in the differential diagnosis [4].

As far as we know, there are no other case reports on the sertraline-induced alopecia. Our study aimed to contribute to the literature and to the physicians in clinical practice.

## 2. Case Report

A 21-year-old, unmarried, high school graduate male patient (E. C. K.) was admitted to our psychiatry outpatient clinic with insomnia, loss of appetite, weight loss, memory impairment, reluctance, malaise, and fatigue. Medical history of the patient revealed no hair loss, no alcohol-cigarette-substance use, and no medical or psychiatric disorders. In the psychiatric examination, patient was conscious, cooperative, and fully oriented with decreased self-care, depressed mood, and impaired vegetative symptoms (initial insomnia and a reduction in appetite and libido). The Structured Clinical Interview for DSM-IV Axis I Disorders (SCID-I) was applied to the patient; no symptoms other than depression were detected [5, 6]. The total score on Beck Depression Inventory was 41. The patient was hospitalized with the diagnosis of major depressive disorder. Sertraline was started at the dose of 50 mg/day. Fifteen days after starting the sertraline treatment, a consultation was requested from dermatology clinic for active hair loss. Thyroid, liver, and kidney function tests as well as levels of vitamin B12, iron and folate, iron binding capacity, serum electrolytes (Na, K, Ca, Cl, and Mg), sedimentation rate, complete urinalysis, total testosterone, DHEA-S, and zinc and copper levels were analysed. Although the examination and treatment processes were not completed, the patient was discharged on his own request. Sertraline was recommended to be used at the dose of 50 mg/day. The patient was told to visit the outpatient clinic a week later. At the outpatients' control visit, hair loss was found to be progressive. Depressive symptoms were found to be decreased with a score of 22 on the Beck Depression Inventory. Since no other cause was found to be responsible for the hair loss, sertraline treatment was stopped and hair loss disappeared in 2 weeks. Another antidepressant drug was prescribed and the patient had no hair loss complaints at his visit 1 month later.

## 3. Discussion

Psychiatric disorders are among the most prevalent diseases [7, 8]. The SSRIs are prescribed by both psychiatrists and nonpsychiatrist clinicians. There are a limited number of studies in the literature reporting SSRIs-induced alopecia. In the study by Spigset [9], dermatological side effects constituted 11.4% of the SSRIs-induced side effects; dermatological side effects were more common with fluoxetine use; and the most common dermatological side effect was rash.

Sertraline differs from other 5-HT reuptake inhibitors in its chemical structure and is a naphthylamine derivative [3]. Sertraline has SSRI a more potent inhibitor effect of dopamine reuptake compared to the inhibition of the norepinephrine reuptake [3]. Sertraline-induced side effects are similar to those induced by the other SSRIs and include headache, dizziness, tremor, sweating, sleep disturbances, dry mouth, gastrointestinal disorders, and sexual dysfunction [3].

Many medications can cause hair loss, although the mechanism has not been fully understood [1]. Psychotropic drugs are considered to cause hair loss by affecting particularly the telogen phase of the hair production [10]. Side effects of these drugs affecting hair are not only limited to the hair loss. They have been suggested to be changing the structure and the colour of the hair as well [11]. Drug-induced hair loss usually occurs within the first 3 months of the treatment [12]. In addition, other antidepressant medications may cause telogen hair loss too. Fluoxetine is the most common SSRI causing hair loss [13]. In a review on this issue, hair loss was found to be present after fluoxetine use in 725 cases and in 6, 7, and only 3 cases using fluvoxamine, paroxetine, and sertraline, respectively [10].

When a patient complains of hair loss due to starting a drug, it is important to be sure that this is a pathological hair loss. Gentle hair pull test can be used to objectively determine the hair loss [14, 15]. Gentle hair pull test is a simple test that can be applied by any physician. In order to gain accurate results patients should avoid washing their hair starting from 5 days before the test. A lock of hair containing an amount of about 50–60 hair roots is held with the first three fingers and pulled gently toward the end. Hairs extracted are counted. If more than 10% of the hair lock (>6) is extracted the test is accepted to be positive and means an active hair loss is present. If it is below 6, it is accepted as the normal physiological hair loss [14, 15]. Our case was hospitalized and this test was applied following the rules and active hair loss was detected.

It is difficult to decide whether hair loss is related to the drug use since there are no available specific methods for a definite diagnosis. For diagnosing drug-induced hair loss, other possible etiologic causes should be excluded and the hair loss and drug starting time or dose changes should be relevant. A reliable way to verify the diagnosis of drug-induced hair loss is the finding of hair loss reduction or disappearance when the drug is stopped and the reappearance of the hair loss reappeared with restarting the drug [11]. However, patients usually do not accept the restart of the same drug [11, 16]. In the present case, sertraline was stopped after the hair loss was observed and was not restarted. The hair loss was considered to be induced by sertraline use, because there was a temporal relationship between hair loss and drug starting time; hair loss disappeared when the drug was stopped and other etiologic causes of hair loss were excluded. Naranjo et al.'s Adverse Drug Reaction Probability Scale evaluates the side effects of the drugs. In this scale, a score of 9 and over, 5–8, 1–4, and 0 is described as certain, likely, potential, and suspicious, respectively [17]. Our case had 9 points and thus hair loss was considered as a certain side effect.

In some case reports, hair loss has been reported to be present after a month of sertraline use [18, 19], while in our case hair loss was seen within the first 2 weeks. This might be related to hospitalizing the patient at the beginning of the treatment. In this way, it was possible to monitor the patient closely and side effects were detected earlier than the patients under the follow-up in the outpatient clinic.

Although the patients in the previous case reports were predominantly female [18, 19], the patient in our case report was male. This might be due to the fact that females take care of their physical appearance; thus they may recognize the physical changes earlier and seek medical help immediately.

Because SSRIs-induced hair loss is rare, clinicians may disregard this side effect. Moreover, many patients may not recognize the hair loss as a side effect of the drug. Patients may not be giving feedback. Because there are a limited number of studies in the literature, the exact prevalence of sertraline-induced hair loss can be detected more clearly by carrying out large-scale studies and increasing feedback levels.
